# Presentation of Asymptomatic Ebstein’s Anomaly: A Case Report

**DOI:** 10.7759/cureus.77227

**Published:** 2025-01-10

**Authors:** Brittani P Kongala, Heather Hare, David W Smith

**Affiliations:** 1 College of Medicine, Florida State University College of Medicine, Tallahassee, USA; 2 Department of Cardiology, Southern Medical Group, Tallahassee, USA

**Keywords:** asymptomatic ebstein's anomaly, congential heart disease, ebstein's anomaly, ebstein's anomaly clinical presentation, surgical repair, tricuspid valve regurgitation, tricuspid valve replacement

## Abstract

This report describes the case of a 58-year-old man with Ebstein’s anomaly (EA) who remained asymptomatic until 55 years of age when he began experiencing acute dyspnea, palpitations, dizziness, and fatigue during exercise. Patients with EA have an increased risk for arrhythmia, right-sided heart failure, and sudden cardiac arrest. This case report highlights the late-onset complications of EA in a previously asymptomatic adult. While this case aligns with the typical collection of symptoms seen in EA, it is unique in that the complications of EA did not develop until the patient was an older adult despite being diagnosed with EA shortly after birth. However, the spectrum of clinical presentation of EA varies greatly from asymptomatic to severe. For patients with asymptomatic EA, this case exemplifies the importance of recognizing the progression and complications of this malformation in older adults to ensure proper interventions are pursued.

## Introduction

Ebstein's anomaly (EA) is a congenital heart defect occurring in one per 200,000 live births which comprises <1% of all congenital heart defects [1-2]. First described by Dr. William Ebstein in 1866, EA is characterized by apical displacement of the tricuspid valve and adherence of the tricuspid septal and posterior leaflets to the myocardium [1-6]. These anatomical features develop due to the failure of the valve to delaminate into individual leaflets during embryological development [1-6]. The characteristic downward displacement of the tricuspid valve into the right ventricle causes atrialization of the right ventricle and tricuspid regurgitation of varying severity [1,2]. Over time, the thinning walls of the ventricular myocardium lead to reduced ventricular contractility and eventually right-sided heart failure [1,2,5,6]. The degree of apical displacement of the septal and posterior leaflets in relation to the anterior leaflet is what makes EA unique from other conditions with regurgitation [2,3,6]. Normal cardiac anatomy requires that such leaflet displacement be < 8 mm per meter square [1,6-7]. Additional cardiac malformations are often seen in patients with EA, the most common being atrial septal defects and patent foramen ovale [1, 5, 6]. Additional malformations pose a risk for right-to-left shunting, and when coupled with the severe tricuspid regurgitation seen in EA, these defects can result in hypoxemia and cyanosis [1,5,6]. 

The severity of EA varies greatly between patients and is most commonly measured using echocardiographic imaging. Severity is determined by comparing the area of the right atrium and atrialized right ventricle to the area of the remaining functional right ventricle and left heart [2]. An increased ratio indicates a greater degree of malformation [2]. Less than 50% of patients with EA will survive to five years of age, with the most common cause of death arising from tachyarrhythmias [3]. Ebstein's anomaly also has a wide range of clinical presentations [7]. Neonates commonly present with cyanosis, cardiomegaly, and congestive heart failure [6]. Older children and adults often experience additional symptoms including palpitations, arrhythmias, exercise intolerance, fatigue, and syncope [5-6].

The causes, risk factors, and genetic contributions of EA are not well understood [3,5]. Most cases of EA are idiopathic, with familial EA being uncommon; however, EA has been observed in those with a family history of congenital heart disease [3]. Cases of EA have been described with a variety of chromosomal abnormalities and mutations [8]. One such connection has been made with duplications on the distal arm of chromosome 15, an area known to be responsible for the early formation of cardiac structures, including the tricuspid valve [1,8]. Other hypotheses include rearrangements in chromosome 11q, mutation of cardiac transcription factor NKX2.5, and involvement with chromosome 9 demonstrated in canines [1-3,9]. Known risk factors for EA include maternal use of lithium or benzodiazepines during pregnancy, maternal tobacco use, second-hand smoke exposure, and a family history of congenital heart defects [1,3,5,10]. In particular, maternal exposure to second-hand cigarette smoke and a family history of congenital heart defects were associated with increased odds of EA in a 1997-2011 population-based case-control study of 135 cases of EA [10]. Mothers of children with EA had 4.1 times the odds of a family history of congenital heart disease, while those exposed to second-hand cigarette smoke had 2.2 times the odds compared to healthy controls [10]. However, no single factor has been attributed as the cause of EA, and it is likely a complex combination of genetic and environmental factors [5].

It is recommended that patients with EA be managed medically with regular evaluation by a cardiologist for the assessment of arrhythmia complications or right ventricular (RV) dysfunction [3,4-6,9]. However, upon onset of symptoms, increasing exercise intolerance, cyanosis, or worsening RV dysfunction, surgical intervention should be considered, including repair or replacement of the tricuspid valve and right atrioplasty [5-6,9]. For patients who are ineligible for surgery, it is recommended that standard heart failure treatment be implemented using digoxin and diuretics [3,9].

We present a case of EA in a previously asymptomatic adult. This case documents a patient with EA through his initial development of symptoms, arrhythmia complications, and eventual tricuspid valve replacement and pacemaker implantation. Despite being cyanotic at birth and diagnosed with EA shortly after, the patient was able to live an active lifestyle without requiring surgical repair of EA until he was 58 years of age. This case of EA is unique due to the delay of complications until much later than typically seen in patients with a history of symptoms as a neonate. Given this variation in disease progression, this case emphasizes the importance of understanding EA progression and complications in older, asymptomatic adults to ensure adequate management and timely surgical intervention when indicated.

## Case presentation

A 55-year-old Caucasian male patient with a significant history of previously asymptomatic EA presented to the emergency department (ED) with acute onset palpitations, shortness of breath, dizziness, and fatigue. The patient stated that his symptoms began after a six-mile bike ride, which he usually does several times per week without difficulty. He denied chest pain, leg swelling, or orthopnea. His medical history consisted of cyanosis at birth with the presence of a holosystolic murmur that was later diagnosed as EA via cardiac catheterization. Past echocardiograms revealed downward displacement of the septal leaflet of the tricuspid valve into the right ventricle, mitral valve prolapse (MVP) with mild regurgitation, and a small membranous ventricular septal defect. The patient had remained asymptomatic from his initial diagnosis as a neonate until his current presentation to the ED at age 55. The patient was known to exercise regularly and reported to have experienced no symptoms during previous activities. He had been managed by a cardiologist since his diagnosis of EA shortly after birth; however, he denied restrictions on his physical activity because of EA. The patient’s medical history was also significant for bradycardia and capsular glaucoma with pseudoexfoliation of the lens for which the patient was taking Alphagan P (brimonidine ophthalmic) 1% ophthalmic solution, Travatan Z (travoprost ophthalmic) 004% ophthalmic solution, and dorzolamide-timolol 2.23%-0.68% ophthalmic solution. He denied any history of coronary artery disease, hypertension, diabetes, or hyperlipidemia.

**Figure 1:**
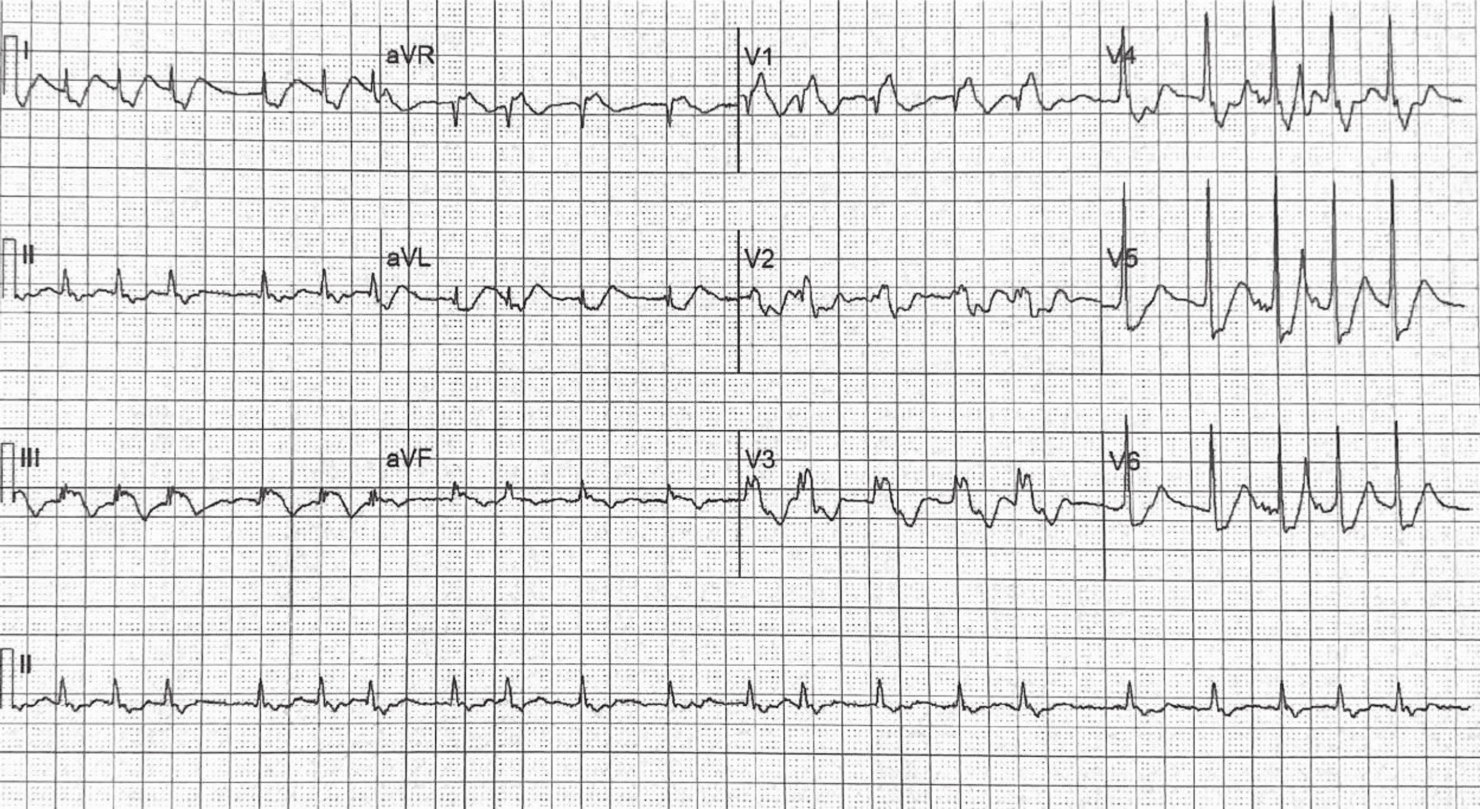
The ECG shows atrial fibrillation with rapid ventricular response and right bundle branch block.

Vitals at the time of cardiology consult in the ED revealed a blood pressure of 115 mmHg/73 mmHg, heart rate of 124 beats per minute (bpm), respiratory rate of 27 breaths per minute, oxygen saturation of 99% on room air, and a temperature of 97.4℉. Cardiac auscultation revealed a widely split S2 with a grade 2/6 holosystolic murmur at the apex with an irregularly irregular rhythm. Chest x-ray (CXR) showed cardiomegaly with trace bilateral pleural effusions. An electrocardiogram was significant for rapid atrial fibrillation and a right bundle branch block (RBBB) (Figure 1). Upon discovery of the atrial fibrillation, he was promptly started on IV heparin and diltiazem. He later underwent direct current cardioversion (DCCV) to restore regular rhythm and was transitioned to Eliquis (apixaban) 5 mg for thromboembolism prevention and metoprolol XL 25 mg for rate and rhythm control. Transthoracic echocardiogram (TTE) from approximately four months prior to symptom onset during a routine cardiology follow-up appointment demonstrated EA, mitral valve prolapse (MVP) with mild regurgitation, severe right atrial enlargement, and a dilated right ventricle with reduced systolic function. At the time of discharge, the patient was instructed to follow up with his cardiologist, and he had an implantable loop recorder placed to monitor his cardiac rhythm.

After his ED visit, the patient followed up with his cardiologist. Six months after the ED visit, a review of the implanted loop recorder showed no prolonged arrhythmias, although occasional irregularity of the heartbeat was noted. The patient reported no chest pain, palpitations, near syncope or syncope, lower extremity edema, or orthopnea. He had also been able to resume his usual physical activity. The ECG showed sinus bradycardia with a heart rate of 46 bpm, a nonspecific intraventricular conduction delay, and nonspecific T wave abnormality.

Three years after the initial presentation to the ED, at age 58, the patient presented once again to the ED with dyspnea, palpitations, dizziness, and weakness while hiking in a remote wilderness. He denied chest pain, edema, or syncope. The ECG showed atrial flutter with a recorded rate of 250 bpm. Isolated ventricular tachyarrhythmias were also noted on a review of the loop recorder that was implanted three years prior. An echocardiogram revealed severe tricuspid regurgitation with severe RV enlargement and normal left ventricular function. He was administered IV amiodarone and converted to a sinus rhythm. The patient was admitted and underwent a second catheter ablation procedure and was started on Multaq (dronedarone) 400 mg and Eliquis (apixaban) 5 mg.

On follow-up with this cardiologist, due to the progression of his arrhythmias, the severity of his tricuspid regurgitation, and the moderate-severe RV dysfunction, his cardiologist recommended that he receive further evaluation and surgical consultation to repair or replace the tricuspid valve and to implant an epicardial pacemaker defibrillator. The patient initially elected to delay surgery in favor of medical management of his arrhythmias. The patient was prescribed amiodarone because of a nonsustained ventricular tachycardia recorded on his loop recorder, but the patient reported side effects of amiodarone, including palpitations, fatigue, diarrhea, and an overall feeling of illness. The amiodarone was discontinued, and the patient then decided to proceed with the surgery consultation two months after his catheter ablation procedure because of his continued fatigue, decreased stamina, and concern for intermittent atrial fibrillation. The surgery included complete repair of EA with replacement of the tricuspid valve, suture closure of the patent foramen ovale, and right atrial reduction (atrioplasty). A pacemaker defibrillator was also placed in his upper abdomen due to the presence of life-threatening arrhythmia prior to surgery. 

**Figure 2:**
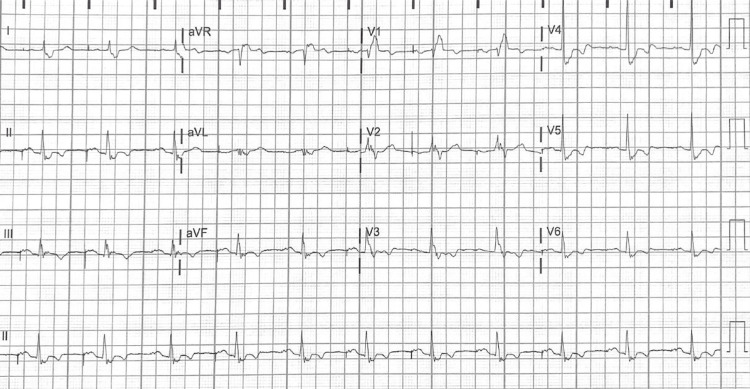
ECG obtained post tricuspid replacement surgery; right bundle branch block with nonspecific T wave abnormality is noted.

Post-surgical ECG can be found in Figure 2. Notably, the RBBB persisted from the earlier ECG but showed an otherwise normal rhythm. Post-surgical TTE revealed an improvement in left ventricular ejection fraction to 65% with a RV pressure of 19 mmHg compared to the pre-surgical ejection fraction of 55% and RV pressure of 30 mmHg on TTE in 2015 (Table 1). The E/A ratio, which is a ratio of early to late diastolic transmitral flow velocity, increased from 0.83 pre-surgery to 1.0 post-surgery. An E/A of <1 indicates an impaired diastolic filling, while healthy individuals typically have E/A >1 [11]. An additional variable, E/E’, is also useful in detecting elevated LV filling pressure. Interestingly, the patient’s E/E’ in 2015 was 11.8 (as seen in Table 1) and reached a level of 20.0 in 2020 post-surgery. The E/E’ of 15.0 measured in 2021 is therefore an improvement despite the elevation from 2020. These changes may have indicated increased LV dysfunction after the operation followed by an improvement in diastolic filling pressure and ejection fraction as of 2021. Missing values in Table 1 represent a disparity in data collected as the two exams were completed at different facilities.

**Table 1:** Comparison of the transthoracic echocardiograms obtained in 2015 (prior to symptom onset and surgery) and the 2021 (post operation). LV: left ventricle; LA: left atrium; RV: right ventricle; RA: right atrium; AO: aorta; AV: aortic valve; PV: pulmonary valve; MV: mitral valve; MIG: maximum instantaneous gradient; E/E’: ratio between early diastolic mitral inflow velocity (E) and mitral annular velocity (E’); E/A: ratio between early diastolic mitral inflow velocity (E) and late diastolic transmitral flow velocity (A).

Transthoracic echocardiogram	2015: Prior to symptom onset	2021: Post-Op
Ejection fraction (%)	54	65
Cardiac output (L/min)	–	5.05
LV diastolic volume (mL)	73.6	112
LV systolic volume (mL)	33.8	39
LA volume Index (ml/m^2)	33	57
LV diameter diastolic (mm)	46	42
LV diameter systolic (mm)	31	28
LV posterior Wall (mm)	8	10
Interventricular septum (mm)	10	11
LA diameter (mm)	49	–
RV diameter (mm)	49	–
RV systolic pressure (mmHg)	29.4	19
Tricuspid valve velocity (m/s)	0.37	–
Tricuspid regurgitant velocity (m/s)	–	1.9
Tricuspid regurgitant MIG (mmHg)	–	14
PV velocity (m/s)	0.99	0.9
AV velocity (m/s)	1.02	1.0
MV velocity (m/s)	0.98	–
E/E’ ratio	11.8	15.0
E/A ratio	0.83	1.0

After recovering from surgery, the patient resumed many of his usual physical activities, including bicycling and walking, without experiencing limitations such as dyspnea, dizziness, or weakness. Unfortunately, despite the surgical corrections, the patient had residual episodes of ventricular arrhythmia in subsequent years and had to undergo an additional ablation four years post surgery. Continued arrhythmias after the surgical repair of EA is a common problem experienced by patients [6]. These arrhythmias were thought to be unrelated to the surgical procedure itself and a result of the persistence of uncorrected aspects of the underlying condition, including RV dysfunction and RBBB.

## Discussion

Given the wide range of clinical presentations and anatomic variations seen in EA patients, classification systems have been developed for consistent grading and improved standardization of diagnosis and treatment despite phenotypic variability on an individual level [12]. While other scales have been described, the 2018 American Heart Association (AHA)/American College of Cardiology (ACC) Guidelines for the Management of Adults with Congenital Heart Disease (ACHD) recommend the use of the ACHD Anatomy and Physiology Classification System. This scale classifies anatomy as grades I-III, ranging from simple to great complexity, and physiological stage from A-D based on symptoms and clinical findings [13].

Cardiomegaly is another classic finding in EA, as was seen in this patient [6]. Ebstein’s anomaly results in an enlarged right atrium because of the downward displacement of the tricuspid valve with a resultant decrease in the functional size of the right ventricle [1]. Atrial enlargement is further exacerbated by the tricuspid regurgitation that occurs secondary to the defective valve [1]. This enlargement, along with RV dysfunction due to ventricular “atrialization,” often results in right heart failure in those with EA [1,2]. A notable detail in this case is that this patient did not develop jugular vein distention (JVD) or peripheral edema despite the moderate-severe RV dysfunction and tricuspid regurgitation. These symptoms may be absent due to increased compliance and dilation of the right atrium, allowing for greater volume accumulation before jugular backflow occurs [2]. The absence of enlarged atrial C-V waves in continuous-wave Doppler has been described in EA with severe tricuspid regurgitation due to the pressure equalization with the right ventricular pressure across the tricuspid valve and large reservoir present in the atrium [1,2, 13, 14, 15].

Morbidity and mortality both greatly depend on the severity of EA measured via echocardiogram [16]. In a study of 72 adult patients >25 years of age, Attie et al. found that the overall median survival was 16.07 years from time 0 to 25 years old (95% CI, 8.64-23.5) [16]. The age of diagnosis is also an important risk factor for survival, meaning that the earlier symptoms appear, the greater the probability of significant progression and mortality [16]. Found in 50% of neonates, cyanosis is a common presentation in EA and poses a higher risk for mortality [1,12,16]. Although it is well-documented that EA has a broad spectrum of severity, the literature indicates that a neonate presenting with cyanosis has a poor survival prognosis [2,12]. In this case, although our patient was cyanotic at birth, he survived into adulthood and remained asymptomatic until the age of 55, not requiring surgical intervention until age 58. The survival rate for EA patients who do not undergo surgical repair is 40% at 20 years, with 8.6% dying from sudden cardiac death within 50 years of birth [1]. Without surgery to repair EA, <5% survive over age 50 [17]. These trends make this patient’s pathophysiology of disease unique and important.

Poor exercise tolerance in EA is associated with severe cyanosis [5]. However, exercise tolerance also depends on the degree of cardiomegaly and oxygen saturation [2]. Müller et al. conducted a study examining longitudinal exercise capacity in patients with EA and found that exercise performance gradually deteriorated in patients who did not undergo surgical correction as they faced progression of arrhythmias and right heart failure [18]. Interestingly, exercise capacity remained stable in those who did undergo surgery [18]. Further research is needed to determine the role, if any, that exercise and an active lifestyle played in delaying the onset of this patient’s symptoms and arrhythmia complications.

Older children and adults with EA more commonly suffer from arrhythmias and hemodynamic deterioration [1,12]. Arrhythmias and abnormalities of the conduction system are frequently noted due to right atrial enlargement and structural interruption of the atrioventricular (AV) node, with 42% of patients exhibiting first-degree AV block [1,2]. A study of 539 patients with EA at the Mayo Clinic found that accessory conduction pathways were present in 13.7% of the sample [4]. A RBBB is also common due to an abnormal bundle branch or fibrosis [2,5-6]. Additionally, anywhere from 25% to 65% of patients experience atrial arrhythmias, including atrial fibrillation and atrial flutter, which may later progress into ventricular tachycardia [1,5]. As seen in this case, the patient began experiencing atrial arrhythmias that progressed to ventricular tachyarrhythmias within a three-year period from the onset of symptoms. At that point in the patient’s disease state, surgical intervention was advised given that ventricular tachyarrhythmias pose the highest mortality risk to EA patients [1,3]. Most commonly patients experience supraventricular tachycardia that precedes atrial involvement. Arrhythmias, including atrial fibrillation, atrial flutter, and ventricular arrhythmias, as seen in this patient's case, are rare and tend to occur in later stages with progressive enlargement of the atrium [5, 19]. It is uncommon that our patient remained asymptomatic up until the development of more progressive arrhythmias. This patient also required the implantation of a pacemaker, which has been shown to be necessary in only approximately 3.9% of patients with EA, most commonly in cases of complete AV block and other sinus node dysfunction [3-4]. In congenital heart disease, epicardial leads are often required due to challenges with transvenous leads in anatomic deviations [3]. Attenhofer Jost et al. reviewed a sample of 15 patients with EA who required pacing assistance and found that 13 cases included the use of epicardial leads, as was the case in our patient [3].

Other indications for surgery include the progression of symptoms such as exercise intolerance, RV dysfunction, arrhythmias, cyanosis, or any incidence of paradoxical embolism [3,5-6,9]. This patient exhibited multiple indications, including exercise intolerance, RV dysfunction, and life-threatening progressive arrhythmia, leading to a recommendation for surgery.

## Conclusions

Ebstein’s anomaly is a rare congenital heart defect with a broad spectrum of severity and clinical presentation. Many patients who are cyanotic at birth require medical management or surgical intervention early in life; however, others may remain asymptomatic until later in adulthood. Even for patients who are cyanotic and symptomatic at birth, they can live asymptomatic for most of their adult lives, only requiring surgical intervention at the presentation and progression of their symptoms. In this patient presentation, the patient was able to live an active lifestyle without limitations to his physical activity previous to his symptom onset at age 55. Surgical repair of EA was required because of arrhythmia progression and worsening RV function and tricuspid regurgitation.
